# Spasmodic Asthma Treated by Chloral

**Published:** 1874-05-01

**Authors:** 


### Gleanings from Our Exchanges.

### SPASMODIC ASTHMA TREATED BY CHLORAL.

*From the London Lancet.*

AT a meeting of the Clinical Society of London, Dr. Theodore Williams brought forward three cases. The first was that of a married woman, aged twenty-three, who came from the Isle of Man, where, during the last nine months, she had suffered from asthma, of so severe a character as to confine her to her bedroom for four months. Various remedies had been tried in vain. On her arrival in town, Dr. Williams did not at first pursue active treatment, hoping that the change of climate might give relief. The fit, however, coming on as usual, chloral was given in twenty-grain doses. After the first dose she fell asleep for an hour; after the second she slept a whole night; and a few more rendered her breathing quite clear. The drug was then omitted, and the patient remained free from asthma for more than a week.

The second case was that of a lad, aged sixteen, who had been subject for six years to attacks occurring once a week and lasting three days. Chloral was given during a severe paroxysm, with the result of causing sleep and immediate relief to the breathing. He remained in the Brompton Hospital free from attacks, in spite of several threatenings of dyspnœa, which were always averted by the timely administration of chloral. The third patient was an unmarried woman, aged twenty-seven, with a history of asthma of two years' standing, the attacks occurring every morning, lasting two or three hours, and often recurring in the forenoon. During a very severe one, which occurred in the Brompton Hospital, a variety of drugs were tried, with little effect. Chloroform inhalation gave some relief, but caused cardiac intermission. Hypodermic injection of morphia did good, but her increasing lividity precluded its continuance. Chloral was then given, in twenty-grain doses, and the first dose induced slumber and easy respiration. The drug was continued, in smaller doses, for upwards of two months, during which time the attacks seldom recurred, and when they did so, were extremely mild. Once the chloral was omitted, and the asthma immediately returned, but ceased on resuming it.

All the cases were complicated by catarrhal symptoms; and in the third case there was considerable emphysema, which diminished during the patient's stay in the hospital. Biermer, of Zurich, had already used chloral extensively in these cases. Dr. Theodore Williams' own experience, founded on upwards of twenty cases, was decidedly favorable to the use of the hydrate of chloral in spasmodic asthma. In only two cases had any untoward symptoms arisen.

---

A NEW OPERATION FOR CLEFT PALATE.—On Saturday, 22d November, Sir William Fergusson, in operating on two patients for the closure of the opening in the hard palate, after the cleft in the soft palate had been closed, adopted a modification of a procedure which is intended to increase the chances of success of the operation. Sir William remarked that in the so-called Langenbeck operation—that is where muco-periosteal flaps are taken from the roof of the mouth and drawn towards the middle line — the proceeding is often unsuccessful from the fact that, after some time, the granulations which are thrown out on the upper surfaces of the displaced flaps contract and separate the union that may have taken place between the pared edges of the flaps. It is true, he observed, that some assert that bony matter is deposited on the upper surface, and that this diminishes the size of the aperture in the osseous palate. But, in demurring to this, Sir Willim said he thought it was hardly possible to strip off healthy periosteum from the subjacent bone. He proposed therefore, as a remedy, that in addition to making the ordinary incisions for the flaps, the hard palate should be split, on each side of the opening, with some sharp cutting instrument, and that the two pieces of bone should be pressed towards the middle line, and the pared edges of the soft tissues then be brought together. By this means the central opening would be closed, but two lateral apertures would be formed. But inasmuch as the lateral openings would be but half the size of the original central one; and as there would be more likelihood of the fractured edges of bone throwing out osseous material for its repair, it was hoped that the prospect of a successful issue would be greatly enhanced.

It remains to be seen what will be the result of this ingenious device; but on the first blush it appears that by its adoption a means is offered of surmounting one of the most obstinate difficulties of plastic surgery.—*London Lancet.*

---

THE PRODROMAL STAGE OF CHOREA. — This period, Dr. Schmitt (*Memorab.*, XVIII., pt. 3, 1873) says, most often escapes the notice of the physician, who in the majority of cases is not consulted until the disease has clearly shown itself. The period is characterized by disturbances, which are confirmatory of the opinion held by Dr. Betz, that chorea is an affection of the central nervous system, particularly of the spinal cord and its membranes. These disturbances are chiefly those resulting from spinal irritation. There is pain on pressure upon the spinous processes, especially in the lumbar and dorsal regions. The patient complains of rheumatic pains in the shoulder and neck; pains in the head are less often mentioned; itching about the anus and nose, which often leads to the suspicion that the patient is suffering from threadworms. There are also symptoms of irritation of the cardiac nerves; general lassitude, unsteady walk; at times there are flashes of light before the eyes; the patient is unable to read or to fix the eyes for any length of time upon one object. The nights are sleepless, disturbed by painful dreams; during the day the patient is subject, without any cause, to severe fits of terror. In one case this stage lasted sixteen days. These symptoms are certainly those of anæmia, depending upon tuberculosis, scrofula, deficient nutrition, or the coming on of menstruation. Dr. Schmitt directs his attention to the treatment of the anæmia by ferruginous preparations and tonics, and has the back rubbed with an ointment containing opium and oxide of zinc. —*Obstet. Jour. of Great Britain and Ireland.*

---

BURNING THE DEAD.—The polite term for this practice is "cremation," or "incremation." Sir Henry Thompson's paper upon it, to which reference was made a few weeks ago, has been translated twice into German; once in Cologne, and once in Gratz, in Austria; in the latter case, with an introductory by Dr. Kœpl, formerly physician to the late King of the Belgians. In consequence of this joint publication, the Communal Council of Vienna has adopted, by a large majority, the proposal of one of its members, to establish in the cemetery the necessary apparatus for cremation, the use of which will be optional and open to all. Following this, the Communal Council of Gratz, which contains a population of one hundred thousand, has decided to consider a like proposal. A veritable agitation of the question has arisen in both places.

In New York city, also, according to a recent dispatch, there are a number of persons zealously in earnest in the effort to introduce the practice of burning the dead, instead of burying them. These gentlemen held a meeting at the office of Dr. Sexton, with a view of perfecting arrangements, either for a large meeting or for some other form of demonstration.—*Phil. Med. and Surg. Reporter.*

Powdered Coal-tar for Wounds.—M. Magnis-Lahens, of Toulouse, adds charcoal to the coal-tar (33 per cent. of the latter), and thus obtains a light and porous powder, which does not irritate wounds, and which is easily washed off with cold water. This combination is a very useful mixture of two antiseptic substances. The charcoal absorbs the gases formed by fermentation, coagulates the albumen, and prevents its decomposition, thus effectually assisting the carbolic acid contained in the coal-tar. Some wounds do not bear powdered applications; for these, 100 parts of the powdered coal-tar should be allowed to macerate for some hours with 400 parts of spirit, and filtrated. The spirit should be of only 18 degrees Cartier, as a stronger would dissolve the resins. As coal-tar principally acts through the carbolic acid it contains, the above-mentioned maceration may be replaced by the following solution: Crystallized carbolic acid, 1 part; spirit (at 18 degrees Cartier), 99 parts. This solution is cheap and very effectual.—*London Lancet.*

Treatment of the Acute Stage of Blenorrhagia by Haschish and Benzoic Acid.—M. Dr. Lamarre (de St.-Germain de Laye) thinks with reason (*Traite des maladies veneriennes*) that there should be no recourse to the abortive treatment of blenorrhagia after twelve or twenty-four hours, when the discharge has become opaline, or purulent, and is accompanied with pain and engorgement behind the navicular fossa. On the other hand, he recognizes the great efficacy of copaiba after the inflammatory period. But we are thus disarmed of any remedy, while the disease is still subacute, for, with the exception of leeches, which few patients will permit to be employed, the baths, applications, camphor, lupulin and bromide of potassium are almost without result.

To supply this deficiency in practice, M. Lamarre has used successfully, for the past seven years, the tincture of haschish in two gramme doses, and benzoic acid, one gramme to be taken in twenty-four hours in some mucilaginous julep.

After six days of this treatment the pain subsides, even during micturition, and patients are better than if they had commenced treatment with cubebs or copaiba.—*Journ. des. conn, med.*—*Gazette des Hopitaux*, March 5. 1874.—*The Clinic.*

Nasal Polypi.—At the last clinical meeting of the Medical Society of London, two very large polypi were exhibited by Mr. Mason. They had hung down behind the velum, and he had taken them with a pair of forceps, and by a slight tug pulled them out. The pedicle was a mere thread. Mr. Mason thought the removal of such growths much less dangerous than is usually believed. Dr. Prosser James concurred in this opinion, and distinguished between these growths and others which had extensive and firm attachments. He dwelt on the importance of rhinoscopy, which, he said, served to detect polypi when they were quite small, and therefore to subject them to treatment. He further insisted on the importance of treatment by local applications, made by the aid of the rhinoscope, by which he had applied both fluids and solids. Such treatment after an operation would also prevent recurrence, and he expressed some surprise that Mr. Mason had not employed this simple preventive measure.—*The Medical Press and Circular*, March 11, 1874.

TREATMENT OF DIPHTHERIA BY CAUTERIZATION.—At a recent meeting of the Medical Society of Nantes, Dr. Thibault related the particulars of an epidemic of diphtheritic angina, in which the employment of cauterizations, with a solution of nitrate of silver, were eminently successful. Dr. Thibault had made use of a solution containing five parts of water to one of nitrate of silver, which he applied to the diseased parts by means of a sponge, after having previously removed the false membranes. These cauterizations, performed with great care and energy, were renewed daily, or every other day, until the membranes became favorably altered, changing from the thick grayish membrane to a soft milk-while one. About three successive cauterizations were employed in each case. Alum was blown on the parts, or used as a gargle, during the intervals. Thus, out of 195 cases of diphtheria observed during the epidemic, there were only 38 deaths, 22 of which were due to the existence of croup. Eight cases of croup recovered; and out of 158 cases of diphtheric angina there were only seven deaths, notwithstanding the extreme gravity of the epidemic, as illustrated by the frequency of consecutive paralysis. It is needless to insist on the importance of the above figures. They show the valuable results of cauterization, which was so warmly advocated by Trousseau and Bretonneau, and which, since, has been much less employed. The use of these cauterizations is indicated, says Dr. Thibault, whenever the false membranes can be easily reached, and, consequently, can be destroyed or modified. They can be easily reached in the pharynx, and their extension downward prevented. It is the difficulty or impossibility of reaching them when they have involved the larynx and trachea which explains the failure of cauterization in croup.—*London Lancet.*

---

Two fatal cases of pyæmia, following the extraction of teeth, have recently been reported by Dr. Langi.

CÆSARIAN SECTION WITH THE ELASTIC SUTURE IN THE WALLS OF THE UTERUS.—The woman upon whom this operation was practiced, was a rachitic patient, excessively deformed, with considerable contraction of the pelvis. She became pregnant at the age of thirty, and the pregnancy, being normal, experienced such insurmountable difficulties in labor, that M. Silvestri (Vicenza) decided to perform Cæsarian section. He operated by the method of Salegres, and the child was withdrawn from the uterus by the presenting arm. An elastic ligature was applied to a severed uterine artery, and four ligatures of the same nature served to unite the walls of the womb. The consequences of the operation were very light, and the patient left her bed on the twenty-fourth day. The child lived.

The author strongly recommends the use of the elastic ligature, which he has thus employed for the first time. By virtue of its elasticity, it follows the uterus in all its movements, and thus maintains its walls in permanent contact.—*L' Osservatore Gazetta delle cliniche*, Nov. 18, 1873. —*Le Progres Medical*, Feb. 21, 1874.

---

LOCAL APPLICATIONS IN NEURALGIA.—*Chlorform.*—Dr. Dupuy speaks very highly of this remedy, used as follows: A pledget of lint, moistened with chloroform, is to be applied to the painful locality, and retained in position a longer or shorter time, depending upon the age, sensitiveness, etc., of the patient, and the part operated upon. Usually, half a minute to five minutes is sufficient, and the application may be renewed from one to a dozen times. Dr. D. states that recent and superficial neuralgias yield to one or two applications, and that even in severe sciatica of long standing, he has never been obliged to make more than twelve.

*Blisters to Apophysal Points.*—The constant presence of such points in neuralgias, as shown by M. Armaingault, has led to the use of blisters, applied in their immediate neighborhood, with very satisfactory results. In cases of facial, intercostal, lumbo-abdominal, and sciatic neuralgias, even when of the most persistent character, and rebellious to other forms of treatment, this plan has been found effectual. — *L'Union Medical*, Nos. 19 and 20, 1874.—*Philadelphia Med. Times.*

INCONTINENCE OF URINE. — Dr. Thomas Kennard, of New York, uses the following ointment in the treatment of this disease: Sulphate of atropia, ten grains; veratria, ten grains; hog's-lard, twelve drachms. By rubbing the perineum three times daily with the ointment, in three cases of paralysis accompanied by incontinence of urine, Dr. Kennard obtained a complete recovery at the end of a few days.—*The Clinic.*

ON a patient suffering of tænia, Dr. Brei, of David's Island, N.Y., proposed to destroy the worm by the use of carbolic acid; but as the solution ingested proved irritating, it was administered in the shape of a pill with licorice powder coated with parafine, to admit of its solution only after reaching the intestines, when, by following with a laxative of rhubarb and jalap, the animal was completely passed on the third day.—*Allg. Wien. Med. Zeit.*

PRESENCE OF LEAD IN THE BRAIN. —M. Trosier (*Le Mouvement Medicale*), while making a chemical analysis of the brain of a patient who had been a worker in lead for more than thirty years, but had never presented any signs of brain disease, discovered well-marked traces of the metal.

